# Characterization of the regulation mechanism of grapevine microRNA172 family members during flower development

**DOI:** 10.1186/s12870-020-02627-6

**Published:** 2020-09-03

**Authors:** Xin Sun, Mengqi Wang, Xiangpeng Leng, Kekun Zhang, Gengsen Liu, Jinggui Fang

**Affiliations:** 1 College of Horticulture, Qingdao Agricultural University, Qingdao, 266109 China; 2 College of Horticulture, Nanjing Agricultural University, Nanjing, 210095 China; 3 Institute of Grape Science and Engineering, College of Horticulture, Qingdao Agricultural University, Qingdao, 266109 China

**Keywords:** Grapevine, VvmiR172 family members, Regulation mechanism, VvAP2, Flower

## Abstract

**Background:**

Grapevine (*Vitis vinifera* L*.*), which has important nutritional values and health benefits, is one of the most economically important fruit crops cultivated worldwide. Several studies showed a large number of microRNAs (VvmiRNAs) involved in the modulation of grape growth and development, and many VvmiRNA families have multiple members. However, the way by which various members from the same miRNA family work is unclear, particularly in grapes.

**Results:**

In this study, an important conserved VvmiR172 family (VvmiR172s) and their targets were set as a good example for elucidating the interaction degree, mechanism, and spatio-temporal traits of diverse members from the same miRNA family. miR-RACE and Stem-loop RT-PCR were used to identify the spatio-temporal expressions of various members of VvmiR172s; together with RLM-RACE, PPM-RACE, Western blot, transgenic technologies, their interaction degree, and regulation mechanism were further validated. The expression of VvmiR172c was significantly higher than that of VvmiR172a, b, and d and showed a positive correlation with the abundance of *VvAP2* cleavage products. These findings indicated that VvmiR172c might be one of the main action factors of the VvmiR172 family in flower development. The ability of VvmiR172c to cleave target genes differed due to divergence in complementary degree with *VvAP2* and expression levels of various members. In VvmiR172 transgenic lines, we observed that 35S::VvmiR172c resulted in the earliest and abundant flowering, indicating the strong function of VvmiR172c. In contrast, the non-significant phenotypic changes were detected in the *VvAP2* transgenic lines. The qRT-PCR and Western bolt results demonstrated that VvmiR172c plays a major role in targeting *VvAP2*.

**Conclusions:**

VvmiR172 up-regulated the expression of *NtFT* and decreased the expression of *NtFLC*. The up/down regulation of VvmiR172c was the most pronounced. The functions of four VvmiR172 members in grape differed, and miR172c had the strongest regulation on *AP2*.

## Background

In plants, miRNAs play important roles in a number of physiological processes, such as growth development and biotic and abiotic stress responses [1–5]; they can regulate the expression levels of their target genes by cleavage. Other miRNAs emerged as promising approaches for plant breeding applications, such as crop improvement strategies [6, 7].

Many plant miRNAs have been identified [8–10], but the majority of these miRNAs remain poorly characterized. Speedy changes in miRNA expression profiles might contribute to plant functions [11–16], and such changes have been described in allotetraploid cotton (*Gossypium hirsutum* L.) [17]. Also, many miRNAs are largely conserved and belong to multigene families [18]. These differences in mature sequences could lead to varieties of target genes type or change the cleavage levels. Multigene families are predicted to target the same or overlapping sets of genes, indicating the possibility of substantial functional redundancy among miRNAs. Other researchers showed miRNA family members that play a specific role in regulation [19–21]. miR156c can participates in the stress tolerance process by regulating its downstream genes, *SPL9* and *DFR*, which take part in this process by influencing the metabolism of anthocyanin [21]. All these abovementioned studies indicated that the action of a full miRNA family was not comprehensively and systematically investigated, and thus, it was difficult to gain insight into the roles of miRNA family.

In grapes, although several studies had identified a large number of VvmiRNAs [16, 22–24], these works focused on the identification of VvmiRNA sequences and their expression profiles at some special tissues/organs. Few studies report research on one miRNA family. Our previous studies revealed that the various members of same VvmiRNA family possessed differential expression profiles and showed the dynamic variation of their expression levels during the grapevine development process [16]. The divergence on the complementary degree between various members of VvmiRNA family and their targets might affect the action degree and regulation mechanism of VvmiRNAs on their target genes. However, the difference in the sequences of various members from the same VvmiRNA family might be an important factor influencing the cleavage degree of miRNAs on their target genes. The differential expression profiles of various members might be another factor affecting interaction. So far, studies that systematically investigated the action degree and regulation mechanism of various members from the same miRNA family on their target genes are lacking. Most previous studies focused on the roles of all miRNA family. Therefore, to more comprehensively recognize the roles of a miRNA family with multiple members, it is necessary and significant to carry out a systematic analysis of each member of a VvmiRNA.

In the previous studies, the experimental validation of miRNAs focused on determining their expressions by Northern blotting and/or RT-PCR. Interestingly, although these two technologies could demonstrate the existence and size of miRNAs, they did not determine their full precise sequences [23–26]. In particular, for miRNA family with multiple members, just the mixture of multiple members’ expressions was detected instead of those of each member. As described above, the variation of diverse members’ sequences from the same miRNA family might affect their complementary degree with corresponding target genes. Thus, these variations might be the action degree and regulation mechanism of miRNAs on their target genes. We developed an integrative strategy of combining miR-RACE and stem-loop RT-PCR together with RLM-RACE, PPM-RACE, and Western blot that could accurately determine the full precise sequences of each member from the same miRNA family. This strategy would also detect the expression.

Levels and cleavage products of each member from the same miRNA family to exactly identify the action degree and regulation mechanism of each member.

The functional exploration of individual miRNA members was meaningful for understanding the function of miRNAs. To discover the regulation mechanism and function of various members from the same miRNA family, VvmiR172 family was used as a typical case investigation in this study. miR172 is an important family that plays critical roles in flower development; it affects the flowering time period, floral organ attributes, and the floral identity by regulating the expression of *AP2-like* genes [27–29]. Currently, several studies showed that most grapevine miRNAs belong to a multigene family, and several members are only different in one to several nucleotides [8, 16, 23, 24]. It is now important to describe the VvmiRNA family members to better understand how miRNAs contribute to grapevine development.

In plants, many miRNAs belong to some multigene families, in which the members are different in mature sequences from individual or multipe nucleotides [18]. The differences in sequences cause variations in matching levels with target genes. Various family members have different temporal and spatial expression characteristics. These differences in mature sequences could lead to varieties of target gene type or change the cleavage levels. Many previous reports were conducted on one member; however, these studies indicate only the characteristics of expression and transgene function [22, 30, 31]. Little attention has been paid to the function and strength of other miRNA family members. Most studies focus on the regulatory function of the whole miRNA family [32–34]. These works did not study each family member. In the present study, miR-RACE, stem-loop RT-PCR, RLM-RACE, PPM-RACE, and transgenic technologies were used to investigate the functions of four miR172 members and the target gene of *AP2-like*genes. This work can elucidate the expression and regulation mechanism on target genes of miRNA family members during grapevine development and provide an efficient strategy that can be used to uncover the role of the different miRNA family members.

## Result

### Diversity of members from miRNA family and their matching degree with targets

**Fig. 1:**
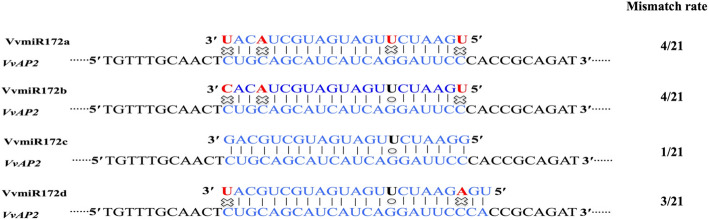
Conserved sequence variation in mature VvmiR172 members and the match between members of VvmiR172 and target genes. Watson–Crick pairing (vertical dashes), mismatch (fork and circles) and G: U wobble pairing (circles) is indicated

Recently, high-throughput sequencing analysis based on small RNA sequencing data suggested that many miRNA families are not a single sequence. A miRNA family is composed of a series of miRNAs with various lengths/sequences. In this study, VvmiR172 family has four members, and sequences of all four members were different (Fig. 1). The alignment analysis of four VvmiRNA family members revealed an overall well-conserved consensus with few variations. The miRNA family has multiple members, and its sequence mostly exists at one or more base differences at the 5′ or 3′ end. The difference of miRNA sequence could lead to the change of complementation with target gene. Among four members, VvmiR172c possessed the least mismatch bases with target gene *VvAP2*, followed by VvmiR172d, VvmiR172b, and VvmiR172a (Fig. 1). Minor variation in the bases of miRNA sequence may alter its ability to regulate target mRNAs.

### Dynamic changes in various members of miRNA family during grapevine flower development

**Fig. 2:**
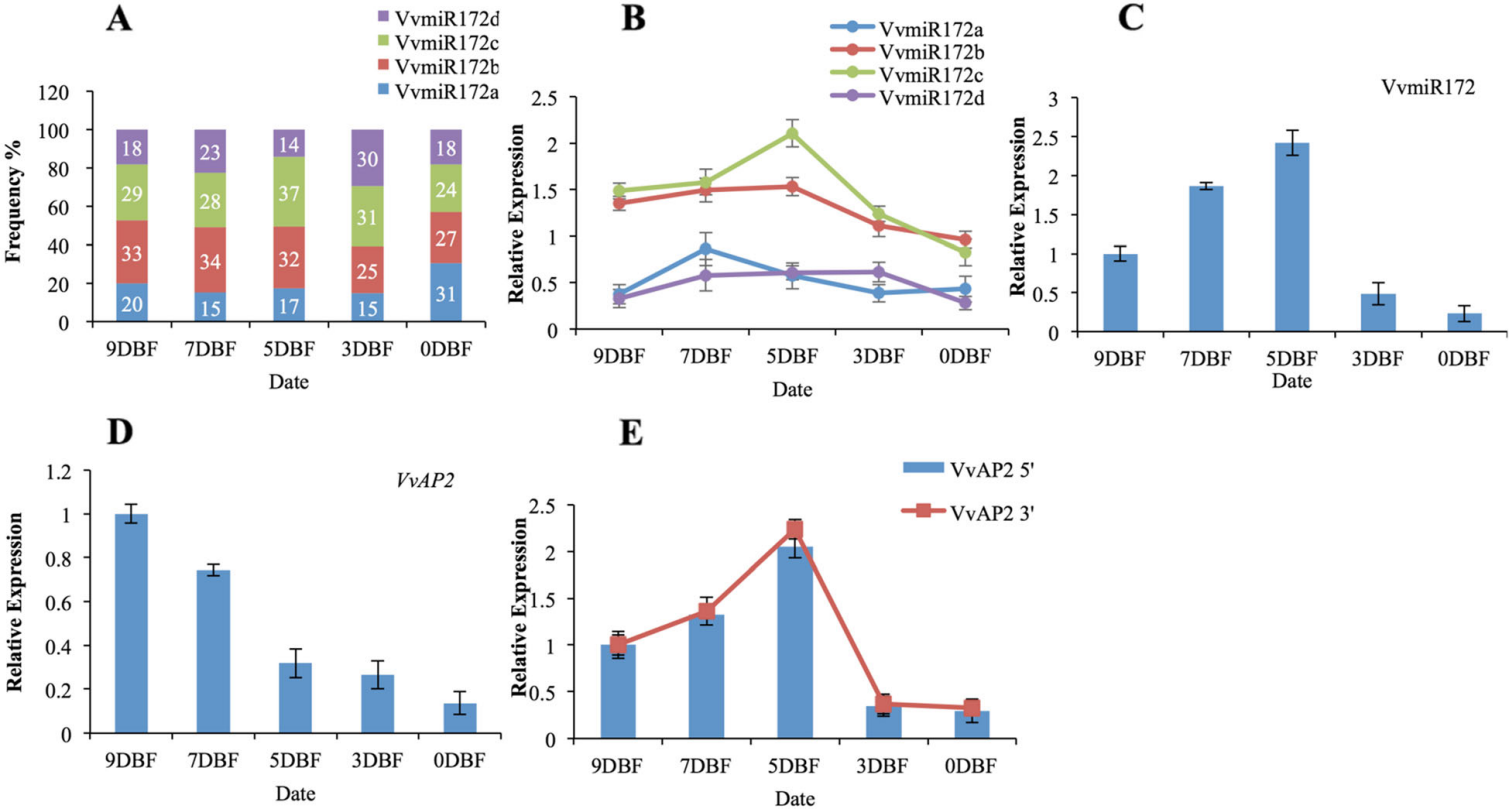
The expression correlation among VvmiR172 family members, target genes, and 3′ and 5′ products of miRNA cleaved target genes. **a** Normalized reads the frequency of VvmiRNA during flower developmental stages. **b** Expression levels of VvmiRNA family members were analyzed by Stem-Loop RT-PCR. **c** The total expression of VvmiRNA family. **d** The expression of target gene. **e** Expression patterns of 3′ and 5′ products of miRNA cleaved target genes by PPM-RACE and RLM-RACE. DBF: days before full bloom

There are many members in the same miRNA family in grapevine that may result in functional redundancy or in a function that has spatial-temporal specificity, but the individual contributions are still unknown. Thus, we examined whether each member plays a particular function in flower development. The miR-RACE and stem-loop RT-PCR was used to analyze the expression levels of different members in the same VvmiRNA family. The expression trends between different VvmiR172 members showed specificity in flower development (Fig. 2a, b, c). However, the proportion of miR172b and c in total expression is relatively higher than the others (Fig. 2a, b); this finding is similar to our previous high throughput results [16]. The trend of VvmiR172c expression was consistent with that of the VvmiR172 family, but the expression of VvmiR172b was downregulated during flower development (Fig. 2a, b, c), thereby indicating that diverse members of VvmiR172 family might possess various regulation mechanisms during grape flower development, and VvmiR172b and c might play the key roles in this process.

### The role of individual miRNA members in regulating target gene expression

Plant miRNAs control gene expression post-transcriptionally through miRNA-mediated cleavage. The spatio-temporal expression profiles of target genes can provide important information in the growth and development of grapevine. Verifying which miRNA members are working on a target and when this event occurs is important to better understand the roles of miRNAs in flower development.

Considering the fact that the individual members of VvmiR172 family showed different transcription by Stem-loop RT-PCR and miR-RACE, we analyzed the roles of each VvmiR172 family members in regulating the target’s expression. The authenticity of the target genes *VvAP2*, for the VvmiR172 family, has been validated [8, 16]. Here we measured the abundance of multiple miRNA members/target mRNA pairs. We found that these cleavage fragments change during flower.

The transcript level of VvmiR172 increased from 9DBF to 5DBF/7DBF and then decreased (Fig. 2a, b, c). The proportion of VvmiR172b and c expression had certain advantages (Fig. 2a, b). *VvAP2* is a typical target gene for VvmiR172 that is involved in regulating flower development. In addition to the expression of VvmiR172b, the expressions of the remaining three VvmiR172 members were positively correlated with the yield of *VvAP2* cleavage products (Fig. 2d, e). This phenomenon could be attributed to the fact that VvmiR172a, c, and d co-participated or only one VvmiR172 member participated in the regulation of *VvAP2* expression (Fig. 2d). To gain further insight into the regulation mechanism of VvmiR172, additional transgenic research is carried out to explore the VvmiR172 family-specific function. In addition, the four members of VvmiR172 family were easily distinguished. Variable control in transgenic detection analysis was probable.

### Functional analysis of each Vvmi172 family members in *Nicotiana tabacum*

**Fig. 3:**
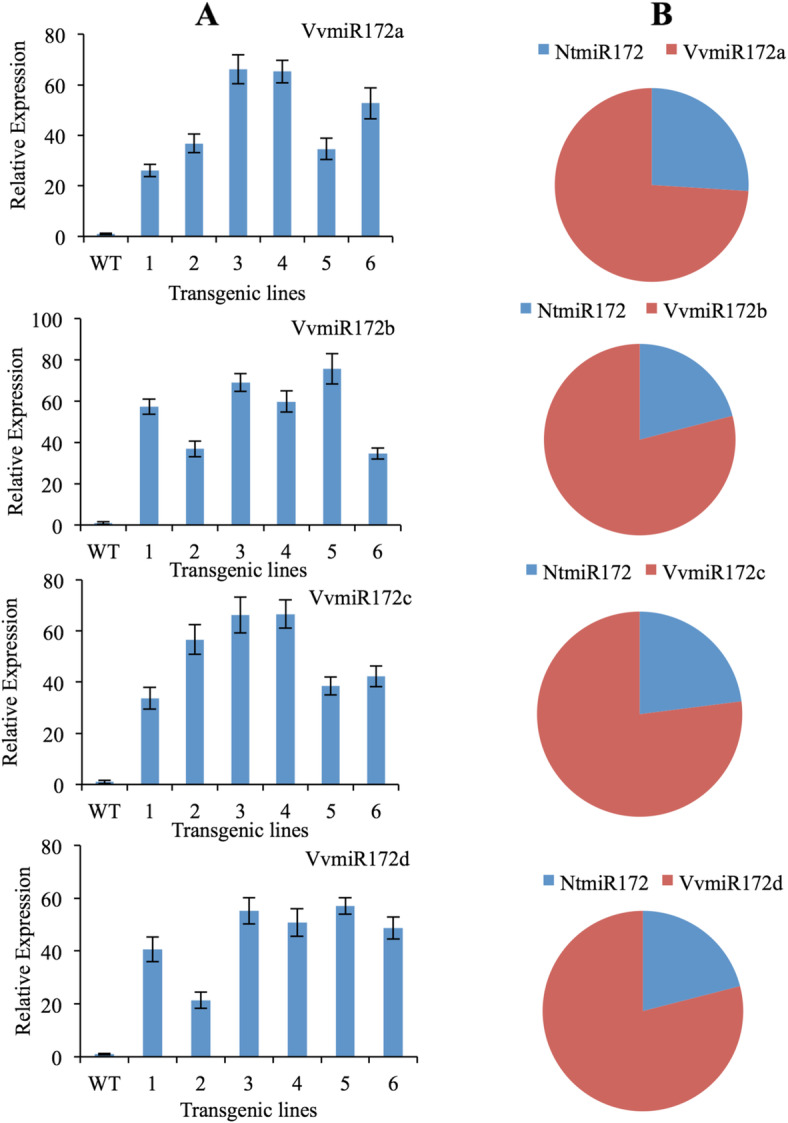
qRT-PCR (**a**) and miR-RACE (**b**) analysis of each VvmiRNA family member levels in transgenic and WT tobaccos. **b** In transgenic plants, the 5′miR-RACE and 3′miR-RACE products were sequenced. The frequency of each member VvmiR172 sequence was statistically analyzed as its percentage in the total miR172 sequences of all the PCR-amplified. The frequencies of all the miR172, considered as their relative expression levels. The red block showed the expression frequencies of VvmiR72 members. Blue Block showed the expression frequencies of NtmiR172

To determine the regulation patterns of target gene AP2 by each VvmiR172 family member, we constructed four over-expression vectors of VvmiR172 family members and transformed them into tobacco (*N. tabacum)*. The accumulation of VvmiR172 in tobacco transgenic lines was analyzed by Stem-loop RT-PCR and miR-RACE. Each VvmiR172 member was detectable in 35S::VvmiR172 lines transformed tobacco (Fig. 3).

**Fig. 4:**
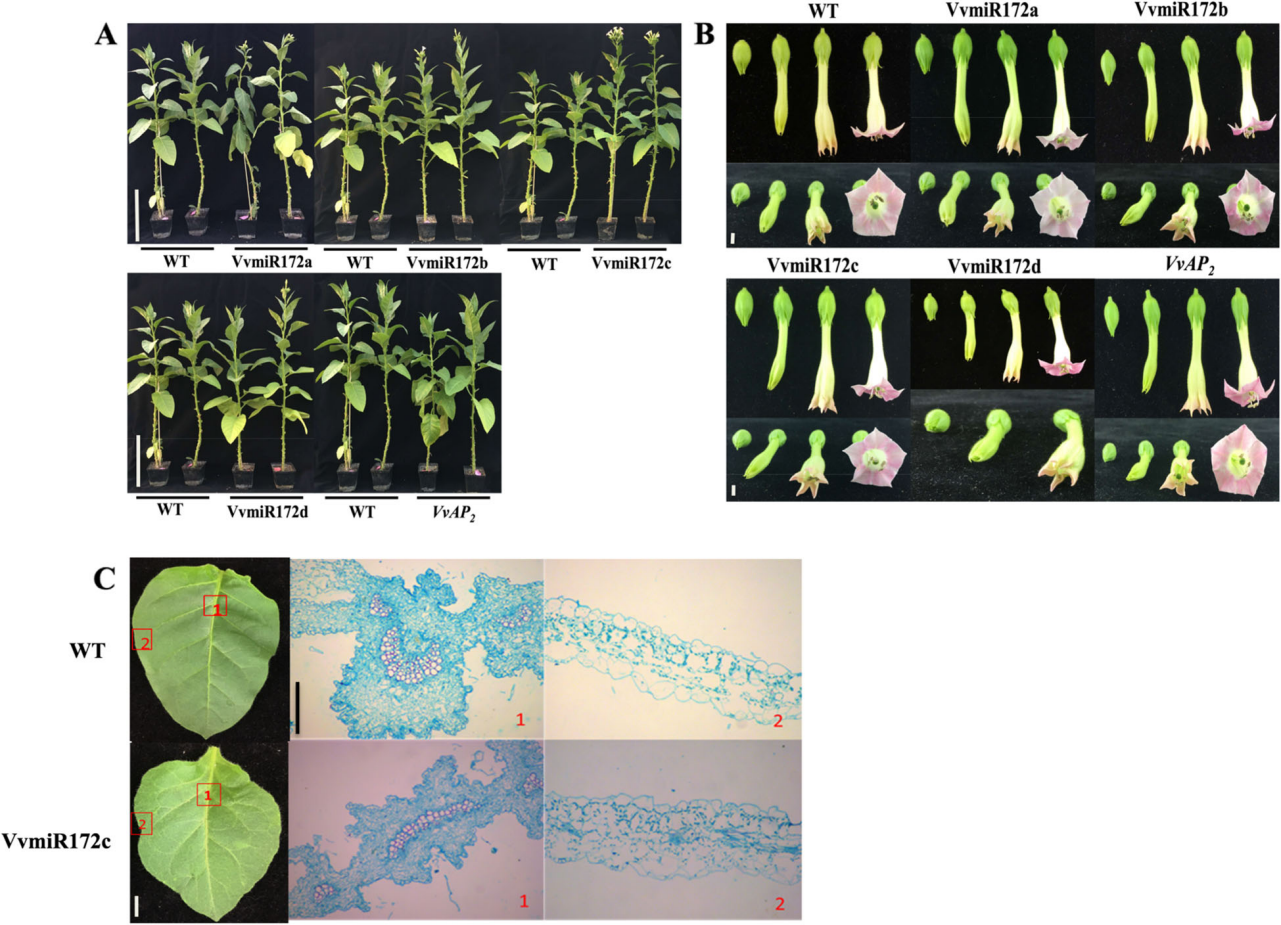
Phenotypic analysis of each Vvmi172 family member transgenic tobacco. **a** The morphoogical phenotype of the transgenic tobacco and wild type tobacco (WT). White scale bar: 25 cm. **b** the flower phenotype of the transgenic tobacco and wild type tobacco (WT). White scale bar: 25 cm. **c** Slice observation of the VvmiR172c transgenic tobacco and wild type tobacco (WT). White scale bar: 25 cm, black scale bar: 90 nm

The function of miR172 was related to the formation of floral patterning [28, 29]. Interestingly, ectopic expression of each VvmiR172 member advanced flowering time (Fig. 4). Floral patterning defects, including sepal-to-petal, was not observed. 35S::VvmiR172c lines comparable with other members’ transgenic lines and wild-type (WT) showed early flowering, and the numbers of nods increased. VvmiR172c leaves were wrinkled, and paraffin sections revealed abnormal vascular tissue cells. The plant height of VvmiR172b, c, and d increased obviously compared with the WT. However, VvmiR172c had higher plant height compared with VvmiR172b and d. Through the phenotypic observation of transgenic tobacco, we can judge that VvmiR172c played an important role in the development of flower and other phenotypic characters of the plant. Interestingly, there were no obvious phenotypic changes in VvAP2 transgenic plants (Fig. 4).

**Fig. 5:**
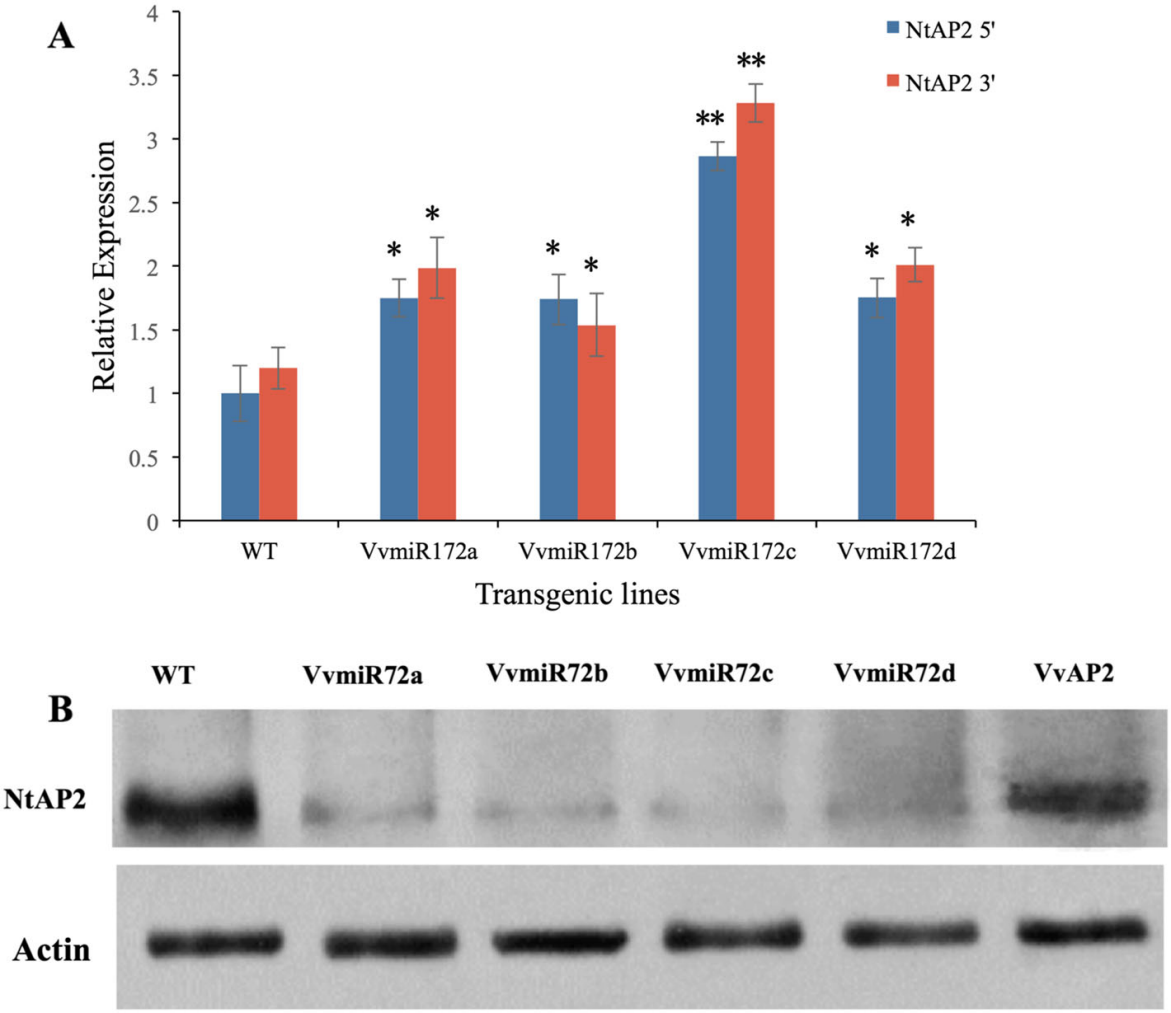
Expression patterns of the cleaved NtAP2 products and NtAP2 protein levels in transgenic and WT tobacco. **a** Expression patterns of 3′ and 5′ products of each VvmiR172 members cleaved target genes from transgenic and WT tobacco by RLM-RACE and PPM-RACE. **b** Immunoblot analysis of NtAP2 protein levels in transgenic and WT tobaccos. Values are the mean of three replicates, and differences with a *P*-value < 0.05 were considered significant. *P < 0.01; **P < 0.001

**Fig. 6:**
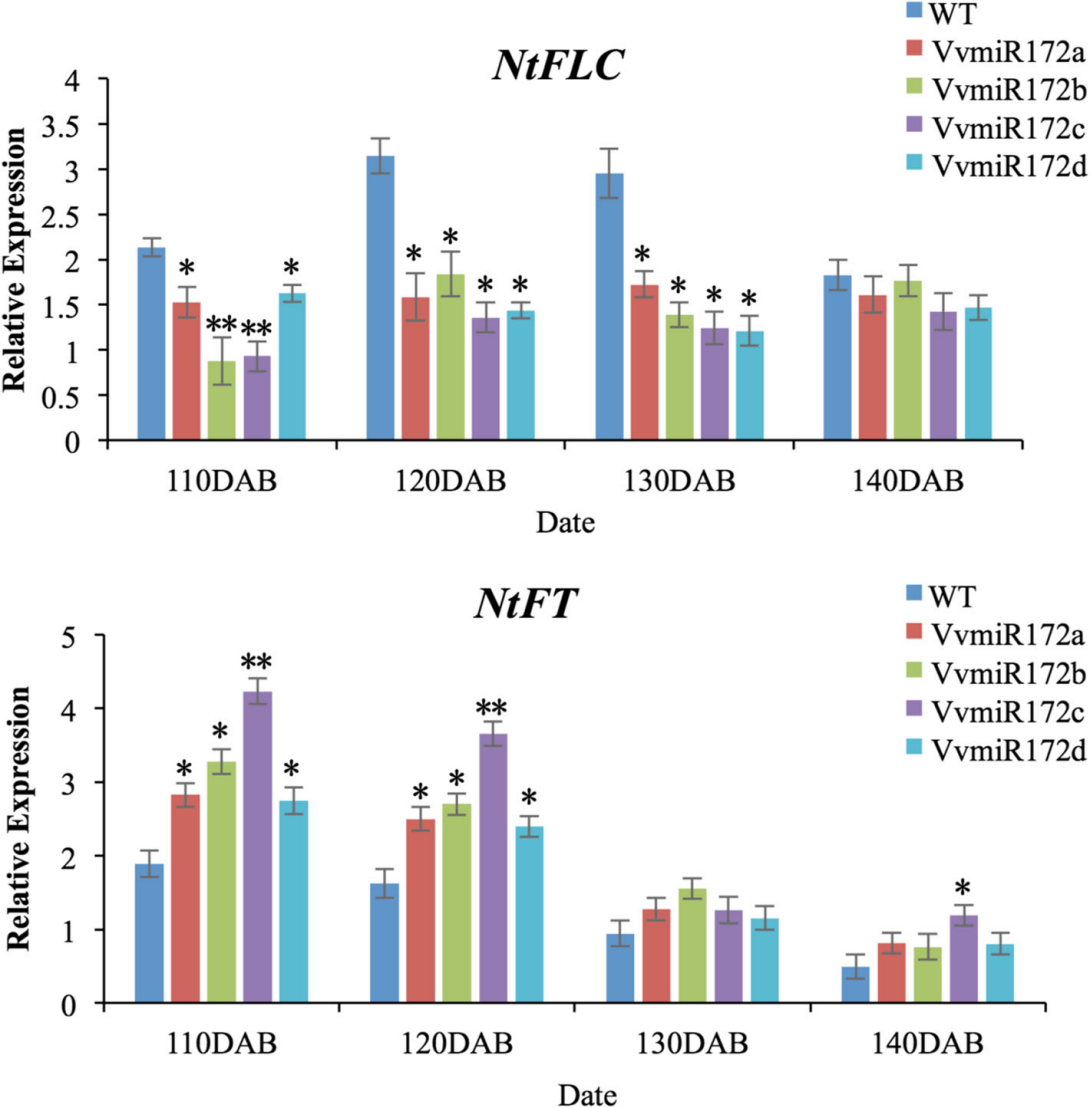
Expression analysis of genes related to flower development (*NtFLC* and *NtFT*) in transgenic and WT tobacco. DAB: days after breeding. Values are the mean of three replicates, and differences with a *P*-value < 0.05 were considered significant. *P < 0.01; **P < 0.001

To further determine the regulatory role of each VvmiR172 family member, the abundance of tobacco *AP2* (*NtAP2*) degradation fragments in individual VvmiR172 members’ transgenic lines was investigated (Fig. 5). The expression of transgenic lines’ cleavage products were higher than those of WT. Although the expression of NtAP2 degradation products in the other three VvmiR172 member transgenic lines were higher than those of WT but still lower than those of 35S::VvmiR172c. The highest cleaved products expression was recorded for 35S::VvmiR172c in all four transgenic lines (Fig. 5a). The *NtAP2* degradation fragments of 35S::VvmiR172a had weak expression in all VvmiR172 transgenic lines. In addition, NtAP2 protein accumulated at a lower level in the each of the four VvmiR172 transgenic lines (Fig. 5b). These results proved that VvmiR172c plays a key role in regulating *AP2*. The transgenic plants had early flowering phenotypes. The transcription levels of some genes related to floral development were detected in transgenic tobacco. The *NtFT* expression in all four VvmiR172 members transgenic tobacco was higher than that of WT tobacco, and the expression of *NtFLC* was lower than that of WT tobacco (Fig. 6). Similarly, the highest expression of *NtFT* was found in 35S::VvmiR172c. However, the difference in *NtFLC* expression between four VvmiR172 members transgenic was not obvious (Fig. 6). Each VvmiR172 member had different functions in flower development, and VvmiR172c plays an important role in regulating *AP2* transcription.

## Discussion

miRNAs function broadly to regulate many aspects of plant development, including flower development, from floral induction to floral organ specification. However, miRNAs belong to a multigene family, and the difference of various members from the same miRNA family only exists in few different nucleotides or single nucleotide in sequences. In model plants, some reports suggested that members of different miRNA families have different levels of expression and have differences or redundancies in their functions. For example, the study of all three miR164 family members mutation indicated that miR164c contributed to a larger extent to the control of shoot development than its two sister miRNAs [19].

In the present study, we observed highly dynamic changes of miRNAs and their family members by Stem-Loop RT-PCR and miR-RACE in grapevine (Fig. 2a, b, c). The expression patterns of VvmiRNA exhibited a certain degree of inverse relationship in expression as expected for target genes, thereby suggesting that these genes might be actively cleaved by VvmiRNA. The expression of VvmiRNA family is not the same as that of any members of the family. There is a positive correlation between the expression of degradation products and the change of miRNA expression, which can be regarded as a standard in evaluating the function of miRNA [35]. The spatio-temporal of different miRNA members could determine the spatio-temporal of target genes. The expression of VvmiR172c/b was higher than that of VvmiR172ad and showed a positive correlation with the abundance of cleavage products, which indicated that VvmiR172c played an important role in flower development (Fig. 2a, b, c). Other reports also suggested that miR172c was an important development regulator [29, 36]. Based on our findings, we propose that different members of the same VvmiRNA miRNA family perform different tasks during grapevine development, with certain key members acting as the main regulators. The abundance of VvmiR172c was the highest, and its ability to regulate *VvAP2* had a great advantage. It had a stronger function of cracking AP2 mRNA. This shows a positive correlation between the expression level of miRNA and its ability to cleave mRNA. Interestingly, the miRNA members with significant regulation had a strong function of cracking AP2 mRNA low mismatching rate with target genes (Fig. 1). AtmiR172 can repress AtAP2 at the level of translation [37]. However, The AtmiR172 have higher mismatches with *AtAP2*, VvmiR172 had lower mismatching rate with *VvAP2*. In previous reports, miR172 possessed the least mismatch bases with target *AP2*, and it could regulate the root and flower development through miR172-induced AP2 cleavage [36, 38, 39]. VvmiR172c had only one error rate with the target gene *VvAP2*, whereas the other three had three or four mismatches with the target gene. The mismatch between miRNA and target genes was low, and the ability to degrade target genes may be stronger.

Since the sequences of four members of the VvmiR172 family differ, they can be distinguished, and the expression of each member in flowers is not obvious. Most importantly, the VvmiR172 matches the tobacco and grape *AP2* exactly the same.

The regulation of target genes by VvmiR172 family members was further investigated by transgenic tobacco. miR172 regulates flowering time and floral organ formation via the degradation and inhibition of *AP2* expression [27–29]. Over-expression of miR172 can lead to early flowering (Table 1, Fig. 4). The observation of tobacco’s ectopic expression of four members of the miR172 family showed early flowering and the increase in the flowering quantity. However, the phenotype of floral organ formation has not been observed. This finding may be because the inhibitory effect of VvmiR172 on *NtAP2* is weaker than that of miR172 in tobacco. Through the transgenic phenotype, we can see that 35S::VvmiR172c had the earliest flowering time and the largest amount of flowering (Fig. 4), which shows that VvmiR172c had the strongest function.

**Table 1:** Comparison of physiological index between transformed and non-transformed plantlets

	Height (cm)	Stemdiameter (cm)	Node number	Internode length (cm)	Ratio of length to width	Flowering time (day)	Flowering amount	Flower diameter (cm)	Flower length (cm)
**WT**	78.36 ± 1.6253	1.03 ± 0.0951	38.33 ± 0.5774	2.74 ± 0.5177	1.91 ± 0.0904	135.2 ± 6.2	71.3 ± 9.07	2.14 ± 0.0547	4.64 ± 0.1141
**VvmiR172a**	79.86 ± 1.0525	1.06 ± 0.1103	37.66 ± 1.5275	2.96 ± 0.3911	1.84 ± 0.0485	126.3 ± 5.6*	68.3 ± 8.51	2.13 ± 0.1141	4.74 ± 0.1517
**VvmiR172b**	83.78 ± 0.8083*	1.12 ± 0.1456	38.67 ± 0.5884	2.82 ± 0.2588	1.89 ± 0.0955	122.8 ± 5.2*	76.2 ± 6.08*	2.11 ± 0.0512	4.61 ± 0.2121
**VvmiR172c**	93.43 + 1.3283**	1.11 ± 0.1731	43.12 ± 1.2358*	3.02 ± 0.2168	2.04 ± 0.0702	118.3 ± 3.6**	87.3 ± 4.35*	2.08 ± 0.0472	4.56 ± 0.1673
**VvmiR172d**	81.73 ± 2.2723*	1.07 ± 0.1723	37.33 ± 1.1547	3.04 ± 0.2511	1.95 ± 0.0874	128.4 ± 4.8*	66.4 ± 8.62	2.02 ± 0.0836	4.58 ± 0.1924
**VvAP2**	76.84 ± 1.3645	1.01 ± 0.2061	38.67 ± 1.3425	2.87 ± 0.3742	1.86 ± 0.0531	143.8 ± 6.5	63.7 ± 7.23	2.11 ± 0.0701	4.62 ± 0.0837

Values are mean ± SE (*n* = 30, *P < 0.05, **P < 0.01). WT: wild-type tobacco. VvmiR172a, VvmiR172b, VvmiR172c, VvmiR172d denote VvmiR172a, VvmiR172b, VvmiR172c, VvmiR172d transgenic tobacco

miRNAs have been heavily studied in grapevine, but VvmiRNAs and their family members are not thoroughly described. We also analyzed the abundance of AP2 degradation products and AP2 protein accumulated in transgenic tobacco plants (Fig. 5). VvmiR172c had the largest cleavage effect on *NtAP2.* No significant phenotypic changes were observed in *VvAP2* transgenic plants. We propose that *AP2* alone cannot play a regulatory role, and over a certain range, its over-expression did not affect the phenotype of plants. The disruption of AP2 caused its function to weaken, and the phenotype caused abnormal flowering time. miR172 could down regulate target genes with flowering inhibitory activity (e.g. TOE1, SMZ, and others), which eventually converged to FT, thereby leading to early blooming [40]. *FLC* can inhibit flowering by weakening the expressions of *FT* and *SOC1* [40, 41].

Based on our data, members of a single family likely perform different tasks during grapevine development. We provide evidence that the expression levels and ability to degrade the target genes of miRNA members within the same family vary in grapevine. From this, we infer that complex mechanisms regulate the expression of these members.

**Fig. 7:**
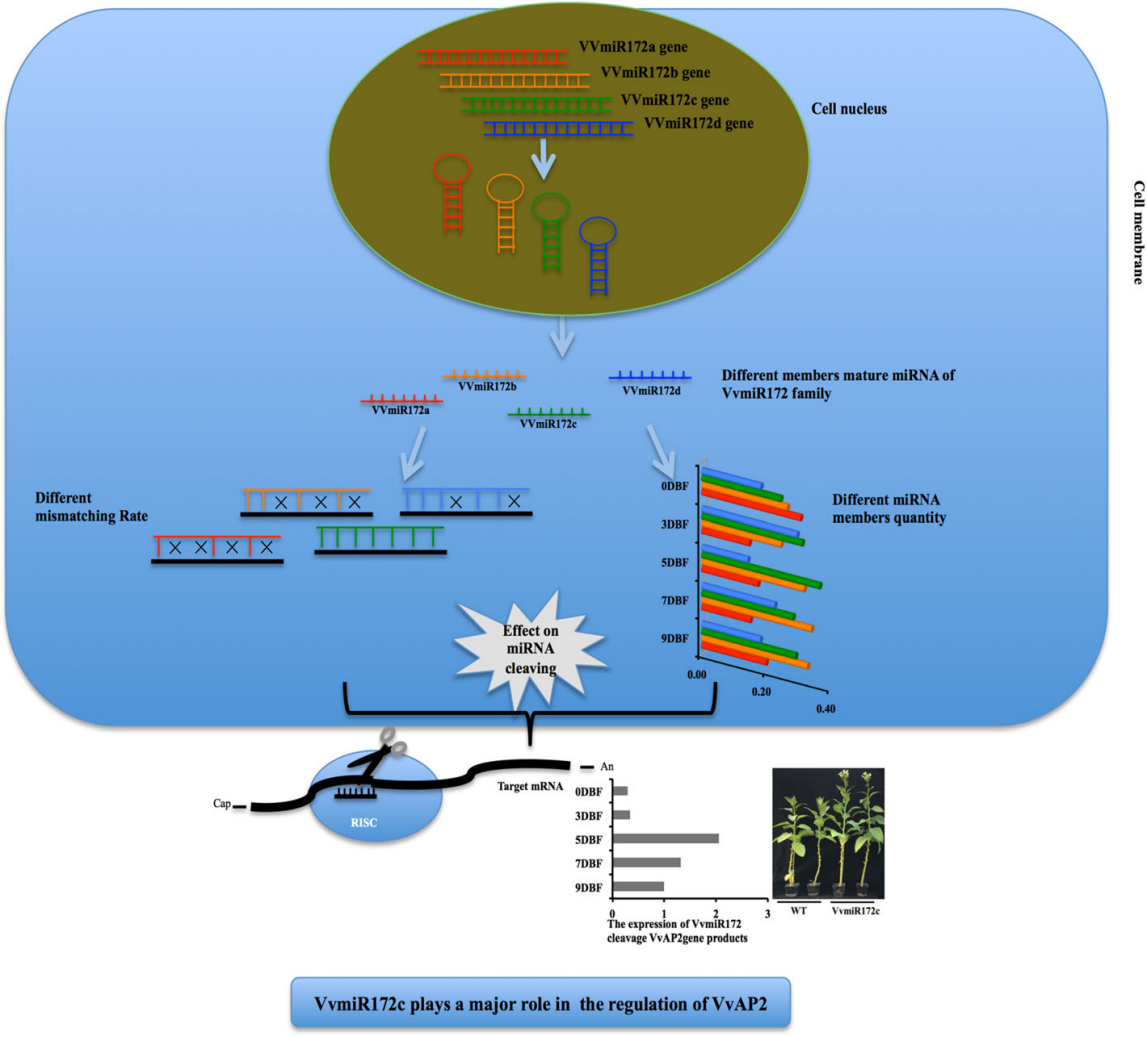
The effect on the cleaving of different miRNA members of the same family. Their expression of VvmiR172 members had great differences, the diversity in sequences and expression levels lead to miRNA regulated target gene VvAP2 ability difference. The mismatch between VvmiR172 and target gene VvAP2 was low, and the ability to degrade target genes may be stronger. Red, yellow, green, blue and black lines indicated VvmiR172a, VvmiR172b, VvmiR172c and VvmiR172d and VvAP2, respectively. The black fork indicated the mismatch between VvmiR172 members with target gene VvAP2

Based on our findings, we propose that the roles of miRNAs and their families’ members extends beyond their initial expression and continues to be active in subsequent developmental stages. We also report great differences in the expression levels of miRNA members (Fig. 7). This work contributes to our understanding of the regulatory network underlying grapevine development.

## Conclusions

VvmiR172 up-regulated the expression of *NtFT* and decreased the expression of *NtFLC* (Fig. 6). The up/down regulation of VvmiR172c was most pronounced. All of the above results showed that the function of four VvmiR172 members in grape differed, and miR172c had the strongest regulation on *AP2*.

## Methods

### Plant material

Plant tissue was collected at each stage of flower development. Tissue was collected from 6-year-old table grapevine ‘Wink’ (*V.vinifera*) grown under standard conditions. The grapevines used in this study were from the Fruit Experimental Farm, Nanjing Agricultural University, Nanjing, China. After collection, samples were frozen in liquid nitrogen and stored at − 80 °C until use. The *Nicotiana benthamiana* was obtain from Laboratory of genetic breeding and genomics of fruit trees (NJAU). All Plant materials were provided free of charge, and were not endangered materials or species.

### RNA isolation, and cDNA synthesis

RNA was isolated from grapevine plant tissue using standard molecular biology protocols and following manufacturer’s instructions, as previously described [5].

### Verification of microRNA expression patterns

First, non-gene-specific products were amplified as previously described, with minor modifications [25]. The 5′ RACE and 3′ RACE clones were sequenced [42], and the relative abundance of each VvmiRNA sequence was analyzed. The results were expressed as relative expression.

### Quantitative stem-loop qRT-PCR of mature miRNA

Grape flower RNA (1 μg) from was reverse transcribed using miRNA-specific reverse stem-loop transcription primers and normalized to *Action* (Supplemental Table S1). Relative expression levels were calculated using the 2^-△△CT^ method and three biological replicates.

### Real-time PCR of total expression of VvmiR172 and AP2

PolyA tailing assay was used to detect the expression of VvmiR172 [43]. Our miRNA-enriched library was used as the template for RT-PCR. To amplify VvmiR172 from reverse transcribed cDNAs, we used the part of the VvmiR172 sequence (GAATCTTGATGATGCTACA) as the forward primer and the R16328 (ATTCTAGAGGCCGAGGCGGCCGACATG) as the reverse primer [23]. The expression of mature miRNA was normalized to that of 5.8S rRNA. The expression of the VvmiR172 target gene AP2 was done as previously described (Supplementary Table S1) [23].

Relative expression levels were calculated using the comparative Ct method with normalization of data to the geometric average of the reference genes by the 2^-△△CT^ method.

### Prediction of targets gene of VvmiR172

miRNAs bind to the protein coding region of their mRNA targets with perfect or near-perfect sequence complementarily. The strategy of VvmiR172 targets prediction was according to the methods reported by Song et al. [25]. These criteria included allowance for no more than two mismatches in the region complementary to nucleotide 1–9 from the 5’end of miRNA, no mismatch for positions 10 and 11, and another two mismatches allowed between positions 12 and 21/24. The number of allowed mismatches at complementary sites between miRNA sequences and potential mRNA targets was no more than four, and no gaps were allowed at the complementary sites.

### A novel RLM-RACE plus PPM-RACE with qRT-PCR cleavage production method

We developed a novel technique for the validation of miRNA target mRNAs that has six steps: (1) library preparation (2), nested RLM-RACE and PPM-RACE, (3) amplicon cloning and sequencing, (4) slicing site detection, (5) qPCR of miRNA-cleaved target mRNA, and (6) cloning and sequencing of products for validation [25, 44].

### Plant transformation and growth conditions

Tobacco (*Nicotianatabacum*) leaf explants were transformed using the *A. tumefaciens* method [45]. The plants were regenerated at 24 °C under 16 h-light/8 h-dark cycles and transformants identified by kanamycin resistance. After root induction, plants were transferred to soil and grown at 25 °C.

### Protein extraction and immunoblotting

Protein wasextracted from leafs of 60 days transgenic tobacco and WT under standard long-day (16 h of light and 8 h of darkness) conditions. The isolation of protein was made by homogenization in 50 mMTris-HCl, pH 8.0, 10 mMNaCl, 0.1 M PMSF, and 0.1 M DTT, with subsequent centrifugation at 13,000 g for 30 min at 4 °C. After electroblotting on a nitrocellulose membrane, protein gel blot analysis was performed using antibodies against NtAP2. Plant Actin was used as loading control.

## Supplementary information

**Additional file 1: Table S1.** Sequences of primer and use.

**Additional file 2.** The orginal figure of western blot.

**Additional file 3.** The coding sequences of VvAP2 and NtAP2.

**Additional file 4.** The sequences of VvmiR172 family members.

## Data Availability

All data generated or analyzed during this study are included in this published article and in its supplementary information files. The materials are available upon request by contacting the corresponding author. Accession numbers of genes in this study: *VvAP2* (MT912541), *NtAP2* (MT912542). The VvmiR172 sequence data could also available from the miRBase (http://www.mirbase.org/summary.shtml?org=vvi) (VvmiR172 accession number: MI0006544, VvmiR172b accession number: MI0006545, VvmiR172c accession number: MI000546, VvmiR172d accession number: MI0006547).

## Abbreviations

miRNA: MicroRNA
AP2: APET ALA2
RLM-RACE: RNA ligase-mediated 5′ rapid amplification of cDNA ends
PPM-RACE: Poly (A) polymerase-mediated 3′ rapid amplification of cDNA ends
miR-RACE: miRNA-rapid amplification of cDNA ends
RT-PCR: Reverse transcription-polymerase chain reaction
qRT-PCR: Quantitative real-time PCR
RT-PCR: Reverse transcription PCR
