# Supplementary figures and images for: Characterization of the regulation mechanism of grapevine microRNA172 family members during flower development
Source: BMC Plant Biol. 2020 Sep 3;20:409. doi: 10.1186/s12870-020-02627-6 (PMC7650276; doi:10.1186/s12870-020-02627-6)

## Slide 1
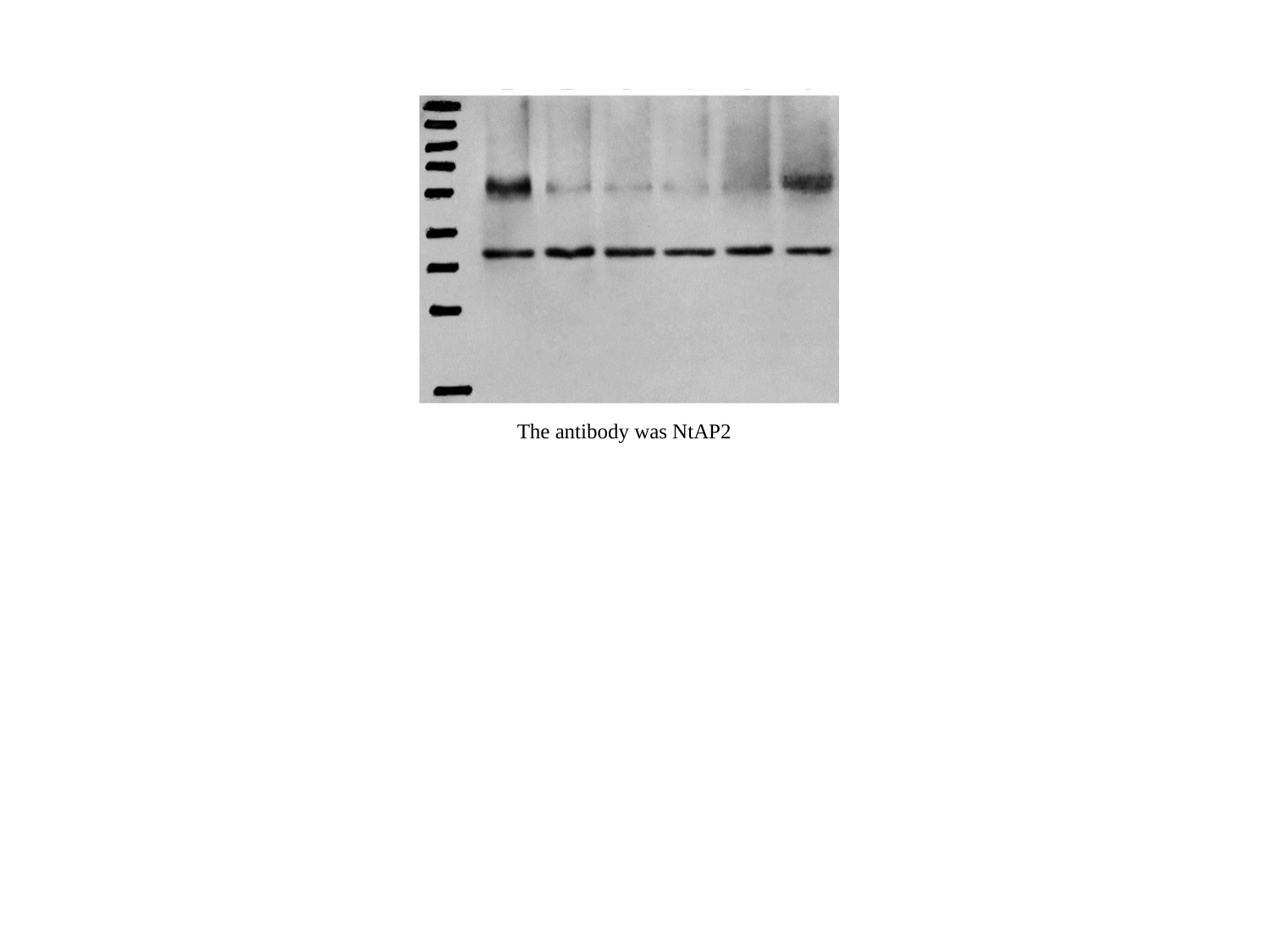

The antibody was NtAP2

## Slide 2
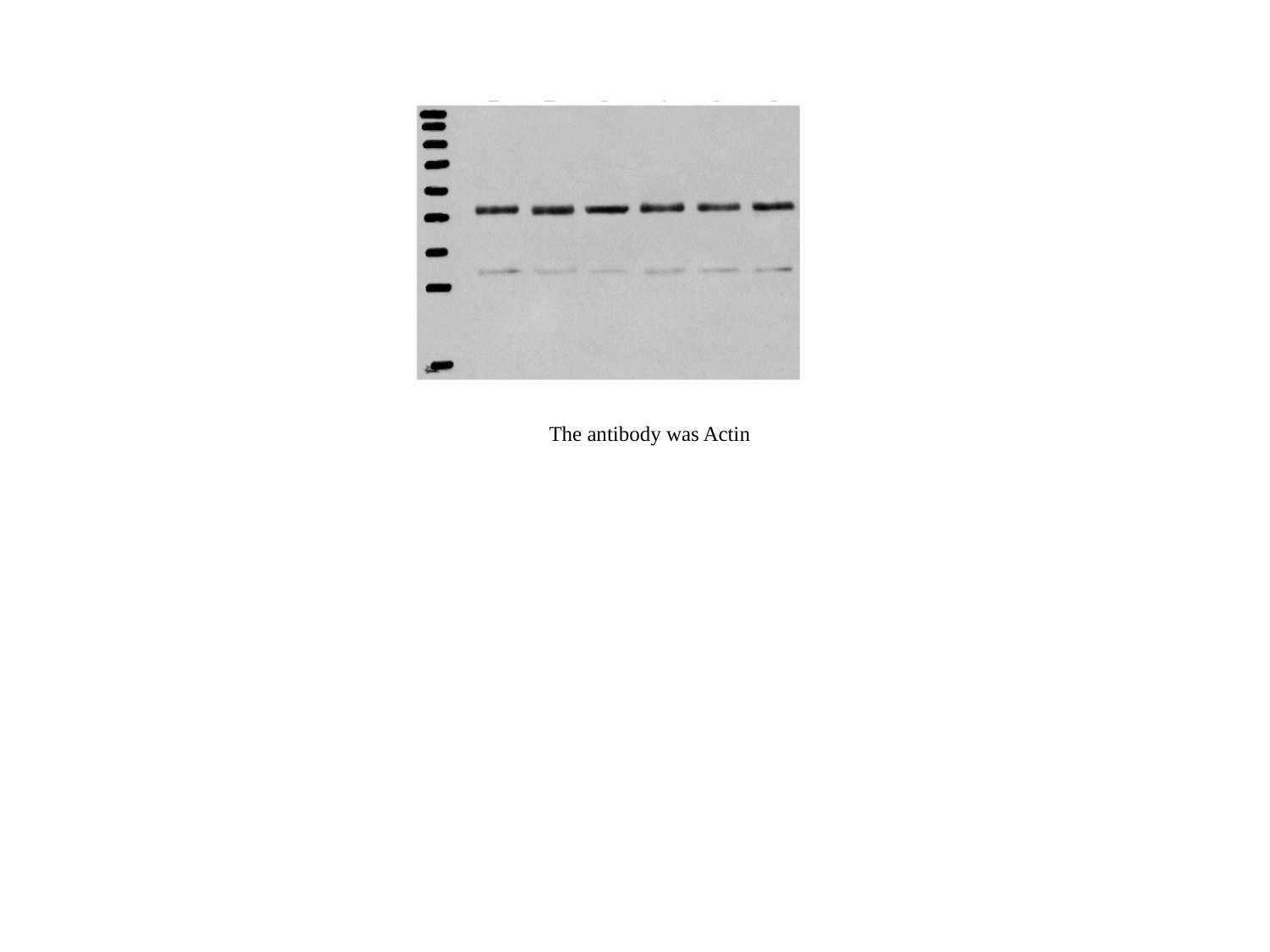

The antibody was Actin

Supplement: Supplementary file 2 — Additional file 2. The orginal figure of western blot. [file 12870_2020_2627_MOESM2_ESM.ppt]
