# Supplementary material for: Characterization of the regulation mechanism of grapevine microRNA172 family members during flower development
Source: BMC Plant Biol. 2020 Sep 3;20:409. doi: 10.1186/s12870-020-02627-6 (PMC7650276; doi:10.1186/s12870-020-02627-6)
Supplement: Supplementary file 3 — Additional file 3. The coding sequences of VvAP2 and NtAP2. [file 12870_2020_2627_MOESM3_ESM.docx]

**The coding sequences of VvAP2 and NtAP2**

VvAP2 (Accession number: MT912541)

ATGTGGGATCTCAACGACTCGCCTGATCAGAGGAGGGACGATGAATCGGAAGGCTGTTCTTCACAGAAGACCTCGGTGGACTCCATCGACGACAAGGGCAAAAGGGTCGGATCGGTTTCCAACGAGAATTCGAGCTCGTCAGCTGTAGTAATCGAGGACGGATCCGATGAAGAAGACGGAGAGAGGGGTCAGAGGAAGCGAAGCAGTAAAATATTCGGCTTCTCAGTGACCCATGAAGATGAGGGTGAGCCACCGGTTACCCGGCAGTTTTTTCCGATGGAGGAGTCGGAAATGGGGACTACTTCCTGCGGTGGTGCTGCGGCCTTCCCTCGAGCTCACTGGGTTGGTGTCAAATTCTGCCAGTCGGAGCCACTCAACACCGCAGGCGTTGCCACCGCCAAGTCACTGGAGGCGTCTCAGCCCTTGAAGAAGAGCCGCCGTGGCCCCAGGTCTAGAAGCTCCCAGTACCGCGGCGTTACCTTTTACCGGAGAACTGGCCGGTGGGAGTCCCACATATGGGACTGTGGAAAACAAGTTTATTTAGGTGGATTTGACACCGCACATGCAGCTGCTCGCGCATATGATAGGGCTGCTATCAAGTTCCGGGGAGTGGAGGCAGACATTAATTTCAGCCTTGAAGACTATGAGGAAGACTTAAAACAGATGGGCAATCTAACCAAGGAAGAGTTTGTGCATGTCCTTCGCCGACAAAGTACTGGTTTTCCGAGAGGAAGCTCCAAGTATAGAGGTGTAACCTTACACAAGTGCGGAAGATGGGAAGCTAGAATGGGCCAATTTTTAGGCAAAAAGTATGTGTACTTGGGCCTGTTCGATACAGAAATTGAAGCTGCTAGGGCCTATGACAAAGCTGCAATTAAATGTAATGGCAAAGAGGCTGTCACCAACTTCGATCCCAGTATTTATGAAAATGAGCTCAACTCATCCGGTAATGCTGCAGATCACAATCTCGATTTGAGCTTGGGTGGCTCCGCATCCAAGCAGAACAATCTGGAATTGGGGGATGACAGCCAAGTTGTTACAATGGATCAGCATTCGGTGGGGATGTCATTTGAAGCTGATTGGCGGAGTCGGGGCTTCAGACCTAAGTTCAACCTGCAGCAGGAGGCGTGTAAAAGCGATGGGGATGCCCTCCGGAGAAATGGATATAATGAAATGGAAACCATGCAGCTTCTGAGCCACACCCACCTACAATCGCCAGCGGCATTAAAGCCAGGTGAAATGTACAGATATGGGCAATTCAGAAGAGCTGGAGAAACCCAGATGCTTCATATACTTCCGCCACAGCTCAGCTCACCAAATTATCAGATTCAGTTTCCAAGCAGCAGTAACGGAGGCCGAATTGCTGTCAACGGAGGAGATCTCTCTCTGGCCACAAACCATCAACAATGGCAATCCGGTCCCCCACAATTGTTTGCAACTGCTGCAGCATCATCAGGATTCCCACCGCAGATGATTAGACCCAACCAACAATGGCCGCAGAAAAATGGGTTCCACTCTCTCATCAGACCCTCCTG

NtAP2 (Accession number: MT912542)

ATGGAGTGTAGAGAAATGTGGGATCTAAACGATTCTCCAGATCGACGAAGGGATGAAAAATCAGAAGAAGGCTGCTCTTCTCCTATAGAGCTAGAGGGCGATGATGAGAAAGGTAAACGGGTCGGATCCGTTTCGAATTCAAGTTCATCGGCAGTAGCTATTGATGATATTTCGGAGGAGGAAGATGGAGAAAAAGGCAAGAAAAAGAGAAGTAGTCCTAGCAAAATATTCGGCTTCTCCGTGGTGGGTCCCGGTAACGACGATGAGGAACAGCCGGTAACCCGTCAGTTTTTTCCGGTTGATGAGTCTGAAACGAGTGCGCCTACCAATGGATCCCCGAATTTTCCCATGGCTCACTGGGTTGGAGTTAAATTTTACCAAACTGAGCCACTTGGCAACACGGGAGCGGGCAAGCCAATGGATGTGGCTCAACAACAGCAGCAGCAGCCTACGAAGAAGAGCCGCCGTGGACCAAGGTCTAGGAGCTCACAGTACCGTGGGGTTACCTTCTACCGGAGAACTGGCCGGTGGGAGTCTCACATATGGGATTGCGGGAAGCAAGTTTATCTAGGTGGATTTGATACGGCACATGCAGCAGCTCGGGCATACGATAGGGCAGCTATCAAATTCCGGGGAGTGGAAGCAGACATAAACTTTAACTTAGAGGATTATGAGTCCGACTTGAAACAGATGACCAATTTAACAAAGGAAGAATTTGTGCATGTGCTTCGGAGGCAAAGTACTGGATTTCCGAGGGGAAGCTCCAAGTATAGAGGGGTGACTTTGCACAAATGTGGTAGATGGGAAGCTAGAATGGGACAGTTCTTAGGCAAAAAGTACGTTTATTTGGGCCTCTTTGATACTGAGGTTGAAGCTGCCAGGGCTTATGATAAAGCTGCCATCAAGTGTAATGGGAAGGATGCAGTTACTAACTTTGATCCTAGCATTTATGAAAATGAACTCAACTCAACTGAATCTACTGATAATGCAGCAGATCACAATCTTGACCTGAGCTTGGGTGGTTCAAGCTCAAAGCAAGGAAACCGAGAAAATGGGGATAATATTAATAGGGGTCAAAATCCTTCATCTATGCAATTTGGTGTTGATTGGCGGCAGCAAGGATTAAGGCCTGAGAAGCAAACTGCTCCAATCGATATGAATGGTCGAAGAAGAGATAAGGGGTACAATGAATCAGAAACGTTGCAGCTCTTGAGCCAGACACACCTACATTCTCCAGTGTCGTTGAAGCCTAATAATAATAGTCAAGTACAACGGTTTGGCCAATTTATGAGACCTGGTGAATCCCATATGATTCAAATGTTTCCACATCAGTTCAGCTCATCAAATTATCAAGTTCAATTTCCAAGCGGCAGCAATGGAGGCAGAATTGGAGCTACAAATGTAGGAGAAATTTCGCTGTCAACAAGCAATGCTTCTTCACAATGGCAATCCAATTATCCTCCTCAGATATTTGCAGCTGCTGCAGCATCATCAGGATTCCCCCAGCAGATAGTAAGACCTCAAAATTGGTCCTCAGAAAATGGCTTCCATCACTCTTTCATGAGACCCTCTAATTGA
