# Supplementary material for: Characterization of the regulation mechanism of grapevine microRNA172 family members during flower development
Source: BMC Plant Biol. 2020 Sep 3;20:409. doi: 10.1186/s12870-020-02627-6 (PMC7650276; doi:10.1186/s12870-020-02627-6)
Supplement: Supplementary file 4 — Additional file 4. The sequences of VvmiR172 family members. [file 12870_2020_2627_MOESM4_ESM.docx]

The sequences of VvmiR172 family members:

VvmiR172a (Accession number: MI0006544):

5’ UGAAUCUUGAUGAUGCUACAU 3’

VvmiR172b (Accession number: MI0006545):

5’ UGA AUC UUG AUG AUG CUA CAC 3’

VvmiR172c (Accession number: MI0006546):

5’ GGA AUC UUG AUG AUG CUG CAG 3’

VvmiR172d (Accession number: MI0006547):

5’ UGA GAA UCU UGA UGA UGC UGC UA 3’
